# New hopes and challenges in targeted therapy and immunotherapy for primary central nervous system lymphoma

**DOI:** 10.3389/fimmu.2025.1438001

**Published:** 2025-02-18

**Authors:** Chuanwei Yang, Xiaohui Ren, Yong Cui, Haihui Jiang, Ming Li, Kefu Yu, Shaoping Shen, Mingxiao Li, Xiaokang Zhang, Xuzhe Zhao, Qinghui Zhu, Xingyao Bu, Song Lin

**Affiliations:** ^1^ Department of Neurosurgery, Zhengzhou University People’s Hospital, Henan Provincial People’s Hospital, Zhengzhou, China; ^2^ Department of Neurosurgery, Beijing Tiantan Hospital, Capital Medical University, Beijing, China; ^3^ Beijing Neurosurgical Institute, Capital Medical University, Beijing, China; ^4^ Department of Neurosurgery, Peking University Third Hospital, Peking University, Beijing, China; ^5^ Department of Pharmacy, Beijing Tiantan Hospital, Capital Medical University, Beijing, China; ^6^ Department of Neurosurgery, China-Japan Friendship Hospital, Beijing, China; ^7^ National Clinical Research Center for Neurological Diseases, Center of Brain Tumor, Beijing Institute for Brain Disorders and Beijing Key Laboratory of Brain Tumor, Beijing, China

**Keywords:** primary central nervous system lymphoma, high-dose methotrexate, targeted therapy, immunotherapy, clinical trial

## Abstract

Primary central nervous system lymphoma (PCNSL) is non-Hodgkin’s lymphoma (NHL) confined to the central nervous system. Most of the patients eventually develop relapsed/refractory (R/R) PCNSL, and the overall prognosis for PCNSL remains dismal. Recently, gene sequencing, transcriptome sequencing, and single-cell sequencing platforms have provided a large amount of data revealing the mechanisms underlying the pathogenesis and drug resistance in PCNSL, including the activation of the NF-κB signaling pathway in tumor cells, tumor heterogeneity, and the immunosuppressive tumor microenvironment. Advances in molecular pathology studies for PCNSL have led to identifying new therapeutic targets and developing novel drugs. New therapeutic strategies, such as creating small molecule targeted agents, immunomodulatory drugs, immune checkpoint inhibitors, and chimeric antigen receptor T (CAR-T) cell therapy, have brought new hope for patients with PCNSL, especially for R/R PCNSL. This review presents recent advances in the treatment of PCNSL, reviews and discusses the efficacy and challenges of targeted therapy and immunotherapy, and provides an outlook on the future development of PCNSL treatment strategies.

## 1 Introduction

Primary central nervous system lymphoma (PCNSL) is a rare, aggressive non-Hodgkin’s lymphoma (NHL) confined to the central nervous system (CNS), including the brain, spinal cord, meninges, and eyes. More than 90% of PCNSLs are diffuse large B-cell lymphomas (DLBCLs) (1, 2). Compared with the systemic DLBCL, PCNSL has a significantly poorer prognosis, with 5- and 10-year survival rates of 29.9% and 22.2%, respectively (3). Approximately 35% to 60% of patients relapse within two years from PCNSL diagnosis, having a poor prognosis (their median survival is only about eight to 18 months) despite aggressive second-line therapy (4, 5). Among patients who survive beyond five years, relapse may still occur in about 50% of cases from five to 13 years after diagnosis. Moreover, approximately 10%-15% of patients receiving methotrexate (MTX)-based combination therapy develop drug resistance and become refractory (6). There is no standard satisfactory salvage therapy regimen for relapsed/refractory (R/R) PCNSL, which contributes to the poor overall prognosis of PCNSL patients. Since the treatment of this group of patients remains challenging, new drugs and therapies are currently being tested in clinical trials to improve the prognosis of PCNSL patients further. In recent years, advances in gene, transcriptome, and single-cell sequencing have helped gain insights into the molecular pathology of PCNSL and explore new therapeutic agents and approaches, the most widely studied of which are targeted therapies and immunotherapies (7). Some of these lines of treatment have been included in the National Comprehensive Cancer Network (NCCN) guidelines. They are recommended for treating R/R PCNSL, frail older patients, and those who cannot tolerate chemotherapy or radiotherapy.

## 2 Progress in molecular pathology studies

Most PCNSL cases are DLBCLs, of which the malignant cells express the pan-B-cell markers CD19, CD20, CD22, and CD79a (8). DLBCL could be classified into germinal-centric and non-germinal-centric types based on the Hans algorithm (9, 10). However, the significance of differentiating pathological subtypes of germinal center B-cells like (GCB) or non-GCB in PCNSL needs to be better understood (11). The results of gene and transcriptome sequencing studies suggest that the oncogenic hallmark of PCNSL is the abnormal activation of the nuclear factor-kappa B (NF-κB) signaling pathway, which regulates B-cell proliferation, survival, differentiation, and cytokine expression (12, 13). The B-cell antigen receptor complex-associated protein beta chain (*CD79B*) and myeloid differentiation factor 88 *(MYD88)* genes are frequently mutated in PCNSL, affecting the activation of the B-cell receptor (BCR) and the Toll-like receptor (TLR) signaling pathways, which in turn triggers the downstream NF-κB signaling pathway and promotes tumorigenesis (14).

In addition, although occurring less frequently, abnormal activation of the PI3K/mTOR signaling pathway, BCL2 and BCL6, can also promote malignant progression in PCNSL (13, 15). Unlike systemic DLBCL, *MYD88* and *CD79B* mutations can occur in non-GCB and GCB types of PCNSL (16, 17). The BCR/TLR-NF-κB axis can be targeted at different levels, either upstream or downstream of the NF-κB signaling pathway, by small molecule inhibitors of Bruton’s tyrosine kinase (BTK) or immunomodulatory drugs, respectively.

Increasing evidence suggests that the tumor immunosuppressive microenvironment also plays a vital role in PCNSL by helping the tumor escape the immune cell surveillance, which promotes tumor proliferation, infiltration, and metastasis (18). Therefore, anticancer therapies should target the tumor and aim to remodel the tumor microenvironment. Tumor-infiltrating lymphocytes (TILs) express a variety of immune checkpoints, including programmed cell death protein-1 (PD-1), cytotoxic T-lymphocyte antigen-4 (CTLA-4), T-cell immunoglobulin, and mucin-domain containing-3 (TIM-3), lymphocyte activation gene-3 (LAG-3), and T-cell immunoglobulin and ITIM domain (TIGIT) (19–21). These immune checkpoints maintain immune tolerance and suppress autoimmunity. High expression of PD-1 is associated with poorer survival (22). In addition to lymphoma cells, tumor-associated macrophages (TAMs) also express programmed death-ligand 1 (PD-L1) (20, 23). TAMs interact with PCNSL cells, contributing to an immunosuppressive environment. Quantification of TAMs may have important prognostic implications (24). In addition, the degree of infiltration of TAMs correlates with interleukin 10 (IL-10) expression in the cerebrospinal fluid (CSF) (25). Thus, IL-10 in the CSF may be a useful diagnostic biomarker in patients with PCNSL (26, 27).

**Figure 1:**
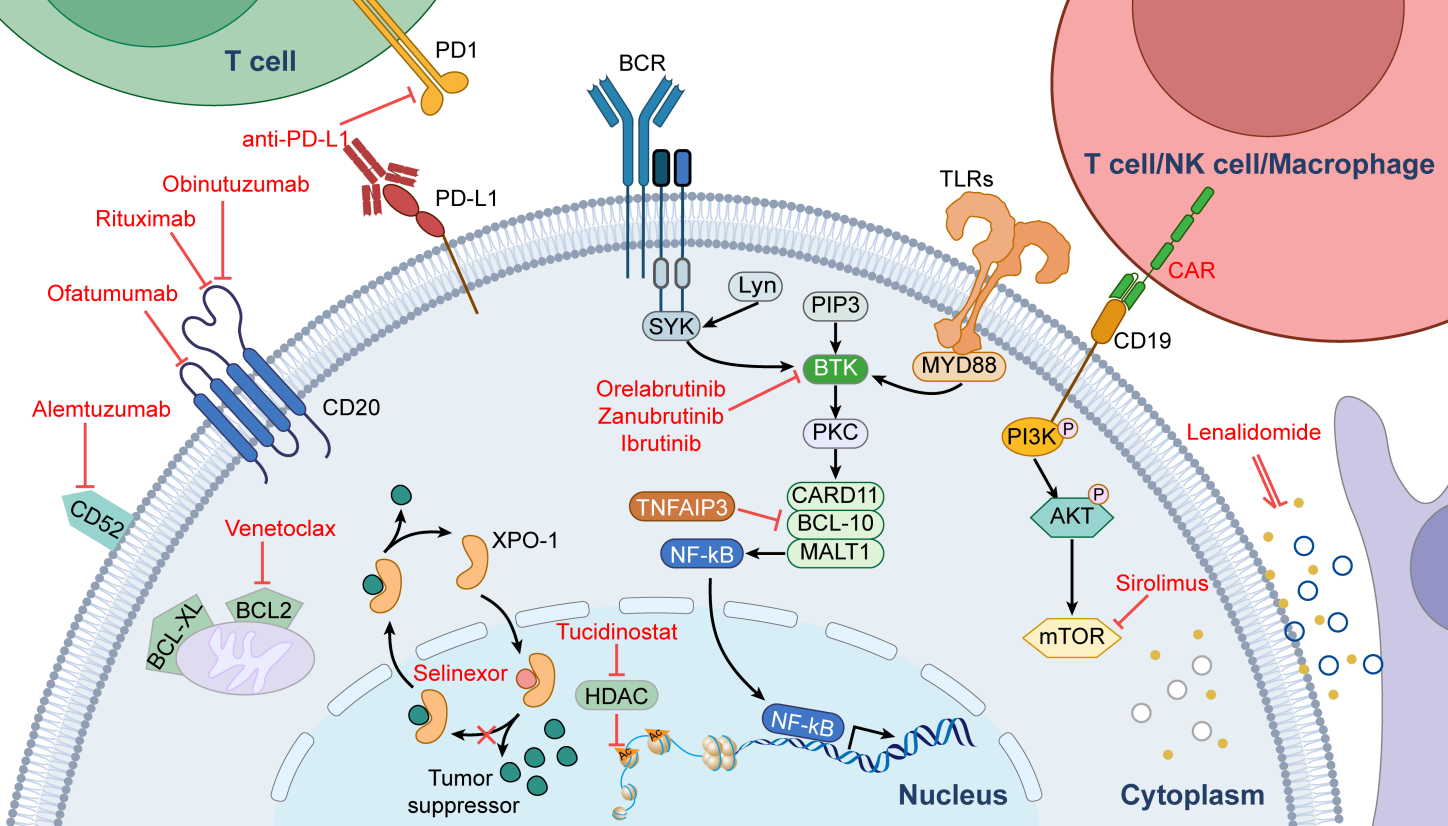
Signaling pathways involved in the mechanisms of PCNSL treatment.

Single-cell sequencing results confirmed that PCNSLs consisted predominantly of tumorigenic B cells, while the remaining cells were mainly immune, oligodendrocytes, and fibroblasts (28). T and myeloid cells accounted for most immune cells. However, the activity of T cells was suppressed, and they became depleted. Compared to peripheral blood, the PCNSL tumor microenvironment was immunosuppressed. Moreover, signaling pathways such as BCR, TLR, and NF-κB were abnormally active in tumorigenic B cells compared to normal B cells in the peripheral blood. To summarize, the above findings suggest that targeted therapies and immunotherapy play an essential role in PCNSL and may have the potential to bring significant breakthroughs in the treatment of PCNSL. **Figure 1** shows signaling pathways involved in the mechanisms of PCNSL treatment.

## 3 Targeted therapy

### 3.1 Anti-CD20 monoclonal antibodies

#### 3.1.1 First-generation anti-CD20 mAb - rituximab

Cluster of differentiation 20 (CD20) is a B cell-specific membrane protein that plays a critical role in B-cell development. It is found on normal B-cells and >90% malignant B-cells, making it an attractive target for therapeutic antibodies (29). Currently, only two prospective randomized controlled trials, IELSG32 and HOVON 105/ALLG NHL 24, have investigated the role of rituximab in PCNSL patients. The IELSG32 trial, which enrolled 227 PCNSL patients and compared treatment regimens of methotrexate and cytarabine with or without rituximab, showed no significant effect on CR rates and overall survival (OS) by adding rituximab. In contrast, the progression-free survival (PFS) difference showed borderline significance (30). Besides, the phase 3 prospective clinical trial, HOVON 105/ALLG NHL 24, which included 200 PCNSL patients and compared the addition or absence of rituximab to a regimen of methotrexate, carustine, teniposide, and prednisone (MBVP), showed no significant difference in PFS and OS between the two groups (24). Moreover, this conclusion still holds true in the study’s recently published long-term follow-up results (31). However, the seven-year results of the IELSG32 randomized trial showed excellent long-lasting outcomes, with a 7-year OS of 37% and 21%, respectively, for treatment regimens of methotrexate and cytarabine with or without rituximab. The superiority of regimens with rituximab over regimens without rituximab suggests a benefit from adding rituximab (32). Long-term results of HOVON 105/ALLG NHL 24 showed that adding rituximab to standard treatment in PCNSL patients did not increase side effects or impact neurocognitive functioning up to 2 years post-treatment (33).

Zhang and colleagues pooled analysis of three prospective randomized controlled clinical studies and eight retrospective studies comparing the effectiveness of rituximab- and rituximab-free regimens for PCNSL revealed that patients on rituximab-containing chemotherapy regimen had a better prognosis than those on rituximab-free regimen (34). However, the inclusion of retrospective data, with its own risk of selection bias and other risks of biases, makes this a less reliable estimate. In conclusion, these results suggest that rituximab has a good safety profile and may be of benefit, but not with high certainty. Although current evidence on the efficacy of rituximab remains controversial, it is recommended by the NCCN guidelines. Because of its considerable molecular weight and low BBB permeability, it is recommended that rituximab should be given before chemotherapy in PCNSL patients with primary treatment or relapse.

#### 3.1.2 New generation anti-CD20 mAb-obinutuzumab

Obinutuzumab is a humanized, glycosyl-engineered anti-CD20 mAb developed in China. The National Medical Products Administration officially approved it in June 2021 in combination with chemotherapy. Obinutuzumab is more active than rituximab in inducing direct cell death (35). Obinutuzumab was not yet approved or guideline-recommended for PCNSL; however, two ongoing clinical trials are currently assessing its efficacy for PCNSL (see **Table 1**). The drug used in combination with obinutuzumab is venetoclax.

**Table 1:** Ongoing clinical studies of obinutuzumab for PCNSL.

Registration No.	Type	Study protocol	Cycle	No. of people	From - To	Key outcome indicators
NCT06175000 (Obsolete Identifiers NCT02498951)	Phase II	ARM 1: C1D1-2 obinutuzumab, D1 obinutuzumab in Cycles repeat every 60 days for 2 years ARM 2: Observation for a total of 2 years.	2 years	30	March 2024 - Sep 2029	1. Overall survival after PR or CR 2. Indicators of neurotoxicity 3. PFS, OS
NCT05211336	Phase I	C1, D1 Nivolumab, D1-14 lenalidomide;	21-day cycle, up to 6 treatment cycles	12	Apr 2022 -Dec 2027	1. Absence of toxicity 2. CR
		C2-6, VIPOR-Nivo (venetoclax, ibrutinib, prednisone, obinutuzumab, lenalidomide)				

C, cycle; CR, complete remission; D, day; OS, overall survival; PFS, progression-free survival. Clinical study data were obtained from the ClinicalTrials.gov database.

### 3.2 BTK inhibitors

BTK is a member of the Tec family of non-receptor tyrosine kinases and is expressed in all stages of B-cell growth (36). BTK promotes upregulation of the downstream NF-κB signaling pathway, which mediates the occurrence and development of B-cell lymphoma, enabling tumor cells to obtain the necessary microenvironment for survival (37). Since BTK is a vital signaling node for PCNSL tumor cell genesis and development, it may be a promising target for PCNSL-targeted therapy.

#### 3.2.1 First-generation BTK inhibitor - ibrutinib

Ibrutinib is an irreversible inhibitor that can covalently bind to cysteine residues at the BTK active site, inhibiting its enzymatic activity. Studies have shown that ibrutinib can rapidly penetrate the blood-CSF barrier to reach central lesions in about 0.29 hours (38). Preclinical experiments in a mouse model showed a brain-to-plasma concentration ratio of 0.7 after oral administration of the drug, and its high concentration in the brain suggests that it may have high efficacy in intracerebral lymphomas (38).

In a phase Ib study, in 18 PCNSL patients with newly diagnosed and R/R PCNSL, including those with *CD79B* and/or *MYD88* mutations, 94% had reduced tumor volume after receiving ibrutinib monotherapy. Out of the evaluable patients, 86% achieved complete remission after combination treatment with ibrutinib, rituximab, temozolomide, etoposide, liposomal doxorubicin, and dexamethasone (39). Moreover, patients bearing *CD79B* and *MYD88* L265P mutations in the tumors had higher response rates to ibrutinib, suggesting that tumors with these particular mutations may be overly dependent on BCR signaling. Notably, aspergillosis occurred in 39% of patients. In another study where ibrutinib was used in combination with chemotherapy, 2% of patients developed severe cases of pulmonary and CNS aspergillosis, possibly because ibrutinib in combination with chemotherapy exacerbated ibrutinib-induced impairment of fungal immune surveillance (40). Grommes et al. conducted a dose-escalation study of ibrutinib from 560 mg/d to 840 mg/d with high-dose methotrexate (HD-MTX) and rituximab until disease progression in patients with R/R PCNSL or secondary central nervous system lymphoma (SCNSL) (41). Out of 13 patients, five had CR, and five had a partial response (PR). The objective remission rate (ORR) was 77%, the median PFS was 4.6 months, and the OS was 5 months. Moreover, this study showed that the ORR of ibrutinib for R/R PCNSL was higher than for systemic R/R DLBCL (77% vs. 25%). During the treatment, the combination regimen had an acceptable safety profile with no grade 5 adverse events and no dose-limiting toxicities. This suggests that a sequential combination of ibrutinib with HD-MTX and rituximab followed by ibrutinib maintenance was effective and well-tolerated (42). Furthermore, Chamoun et al. retrospectively analyzed the clinical data of 14 patients with R/R PCNSL and SCNSL, all of whom had received prior HD-MTX-based combination chemotherapy with ibrutinib monotherapy. Patients with PCNSL showed an overall remission rate of 50%, with two patients having a CR lasting more than eight months and a moderate incidence of adverse events. Reported adverse events included neutropenic fever (n=1), diarrhea (n=1), and peritumoral hemorrhage after failure of ibrutinib (n=1) (43). Thus, the NCCN guidelines recommend ibrutinib for the treatment of R/R PCNSL (44).

#### 3.2.2 New generation BTK inhibitors

Despite having selectivity for BTK, ibrutinib can also bind, with various affinities, to other essential kinases such as EGFR, ITK, JAK3, and HER2 (45). This can lead to off-target effects and undesired side effects, such as bleeding or atrial fibrillation, which to some extent might be treatment-limiting. Next-generation BTK inhibitors have been developed in response to these disadvantages, aiming to improve selectivity, reduce toxicity, and overcome drug resistance. Currently, the main new-generation BTK inhibitors are ibrutinib, acalabrutinib, zanubrutinib, and orelabrutinib (46, 47). Orelabrutinib, a potent BTK inhibitor independently developed in China, has a high selectivity for BTK and nearly 100% BTK occupancy at an oral dose of 150 mg daily. It was authorized for marketing on December 25, 2020, for the indication of chronic lymphocytic leukemia (CLL)/small lymphocytic lymphoma (SLL)/mantle cell lymphoma (MCL) with at least one prior therapy but is not currently approved for the PCNSL indication (48).

Our single-center retrospective analysis (49) revealed a partial effect of orelabrutinib monotherapy for R/R PCNSL, with an optimal efficacy for PR only. However, when combined with RMT (rituximab, methotrexate, and temozolomide) and lenalidomide, an overall response rate of 86.7% and a CR rate of 73.3% was achieved, with grade 3 liver function abnormalities and fatigue being the most common adverse reactions (no grade 4 adverse events or treatment-related deaths occurred). Moreover, we found that in patients where ibrutinib treatment was effective, abnormal activation of the NF-κB signaling pathway was observed. At the same time, dysregulation of transcription factors was present for the ineffective treatment. Additionally, we observed that patients with genetic mutations in the BCR, TLR, and NF-κB signaling pathways benefited from the ibrutinib combination therapy, with some of them achieving and maintaining CR until the last follow-up in May 2022. Our analysis indicates that orelabrutinib can inhibit tumor growth in PCNSL, which depends on activating the BCR and TLR signaling pathways. Importantly, genetic sequencing can provide valuable information on the effectiveness of BTK inhibitors in patient populations.

### 3.3 PI3K/AKT/mTOR signaling pathway inhibitors

The phosphatidylinositol 3-kinase (PI3K)/protein kinase B (AKT)/mammalian target of rapamycin (mTOR) pathway regulates protein synthesis by integrating signals from growth factors, hormones, nutrients, and energy metabolism to regulate cell growth and proliferation (50). PI3K/AKT pathway activation has been detected in many PCNSL samples. PI3K/AKT signaling plays a vital role in mediating the survival of mature B cells. Moreover, PI3K activation is essential for the survival of CD79B mutant cell lines. The studies of BCR signaling blockade in B cells showed that BCR-deficient mature B cells could survive through the downstream PI3K signaling pathway. mTOR, a serine/threonine protein kinase, belongs to the PI3K-related kinase family and is an essential downstream target of the BCR signaling pathway (50).Therefore, targeting key components of PI3K/AKT/mTOR signaling may represent a potential treatment for the PCNSL (50).

Grommes et al. reported a phase II clinical trial with buparlisib monotherapy (PI3K inhibitor) being only 25% effective in patients with R/R PCNSL (12). Results from four patients showed that buparlisib levels in the CNS were significantly lower than the half-maximal inhibitory concentration needed to induce cell death in lymphoma cell lines. In addition, only one patient achieved PR but later developed psychiatric symptoms, leading to study termination. A limited BBB penetration of buparlisib may be the possible reason for the low response rate and, ultimately, clinical treatment failure.

Fimepinostat (CUDC-907) is a small molecule inhibitor of PI3K that downregulates MYC protein levels through inhibition of PI3K-mediated ubiquitination in both solid tumors and hematologic tumor cell models. In phase I and II clinical studies, the efficacy of treatment with CUDC-907 was evaluated in patients with R/R DLBCL, with ORR of 18.1%, a CR rate of 8.6%, and a PR rate of 9.5% after one cycle of treatment, compared to the patients bearing *MYC* mutation with ORR of 23.3%, a CR rate of 13.3%, and a PR rate of 10% (51). These results suggest that CUDC-907 holds therapeutic promise for patients with R/R DLBCL with *MYC* mutations and may provide similar benefits in PCNSL, for which no study results are available.

In a multicenter phase II study in Germany, the efficacy and safety of the mTOR inhibitor tesilomox for R/R PCNSL were evaluated (52). The treatment was effective in 54% of patients, but the median PFS was limited to 2.1 months, and the median OS was 3.7 months. Eight patients (including three unconfirmed cases of CR) achieved CR and 12 PR. The most common toxic side effects were hyperglycemia, myelosuppression, infections (mainly pneumonia), and fatigue. Five patients died from treatment-related complications, and the treatment-related mortality rate was 13%.

In summary, PI3K inhibitors have limited efficacy as monotherapy in treating PCNSL. However, they can enhance the effectiveness of chemotherapy and immunotherapy, thereby potentially improving the prognosis of cancer patients. Overall, PI3K inhibitors are a valuable addition to the treatment of PCNSL and deserve further study to optimize their use in clinical practice (53, 54).

### 3.4 BCL-6 and BCL-2 inhibitors

The proto-oncogenes B-cell lymphoma-2 (*BCL-2*) and B-cell lymphoma-6 (*BCL-6*) are transcriptional repressors that belong to the BCL-2 anti-apoptotic family. They regulate genes mainly related to cell activation, differentiation, and proliferation (10). Studies have shown that high expression of *BCL-2* in PCNSL is associated with poor prognosis (10).

Since BCL-6 inhibitors (e.g., compound 79-6) bind to BCL-6 with weak affinity, their clinical applications are limited (55). A small molecule venetoclax, a highly selective BCL-2 inhibitor, has been approved by the FDA to treat CLL. A phase I clinical study (NCT04073147) explores the treatment efficacy of a combination of venetoclax and anti-CD20 mAb obinutuzumab in R/R PCNSL. The ratio of venetoclax concentrations in the CSF to plasma in human subjects was approximately 1:300, indicating poor penetration through the BBB and suggesting that its efficacy may depend on the BBB disruption (56).

### 3.5 Histone deacetylase inhibitor - chidamide

Chidamide is an oral subtype-selective HDAC inhibitor developed independently in China as a new drug. It was approved by the China Food and Drug Administration in 2014 to treat R/R peripheral T-cell lymphoma (PTCL). The results of preclinical studies suggested that chidamide could cross the BBB (57). This has been confirmed in a clinical study which reported that the CSF to plasma drug concentration ratio was 2.51% ± 2.66% (range 0.15-8.82%) in 10 patients with lymphoma without CNS involvement after four hours of oral administration of chidamide (58). By selectively inhibiting relevant HDAC isoforms, chidamide induces epigenetic alterations by targeting multiple signaling pathways, inhibiting the tumor cell cycle, and inducing apoptosis. This modulates cellular immunity, inducing and enhancing the expression of natural killer (NK) cells and antigen-specific cytotoxic T lymphocytes (CTL) mediated tumor-killing effects, indicating that chidamide may play a role in PCNSL (59, 60). Currently, there is one ongoing clinical trial of chidamide for central nervous system lymphoma (CNSL) (see **Table 2**).

**Table 2:** Ongoing clinical studies of chidamide for CNSL.

Registration No.	Type	Study protocol	Cycle	No. of people	From - To	Key outcome indicators
NCT04516655	Phase II	D1-14: ertapenem 20 mg b.i.d orally; D1: rituximab 375 mg/m^2^ intravenously; D2: methotrexate 3.5 g/m^2^ intravenously.	21-day cycle, six cycles in total	51	Sept 2020 -Aug 2023	Complete response rate

D, day; b.i.d, twice a day. Clinical study data were obtained from the ClinicalTrials.gov database.

### 3.6 Other targets

Other potential therapeutic targets for PCSNL are being explored. Loss of CDKN2A has been observed in PCNSL and may be targeted by cell cycle protein-dependent kinase inhibitors (61). A small prospective study of abemaciclib for CNSL is ongoing (NCT03220646). Selinexor, an inhibitor of nuclear export protein 1, accumulates tumor suppressor proteins in the nucleus, leading to tumor cell death. It has been approved for the treatment of refractory multiple myeloma and relapsed systemic diffuse large B-cell lymphoma (62). Preclinical data suggest that selinexor may have a synergistic effect with ibrutinib, providing a rationale for the future clinical trials of PCNSL (63).

## 4 Immunomodulators

Immunomodulators are a class of drugs possessing pleiotropic immune-modulation properties that can interfere with the growth and survival of aggressive lymphoma through multiple mechanisms of action, including altering tumor microenvironment and stimulating effector cells, such as cytotoxic T cells and NK cells (64). Immunomodulators can also inhibit NF-κB and thus block the PI3K/AKT pathway and are promising agents against PCNSL (64).

### 4.1 Lenalidomide

Lenalidomide, a second-generation immunomodulatory drug, and derivative of thalidomide, has multiple anti-tumor anti-tumor properties, such as stimulation of T-cell and NK cell expansion, which interferes with the growth and survival of aggressive lymphomas through various mechanisms. Moreover, it can have a direct cytotoxic effect associated with PCNSL through the antagonism of interferon regulatory factor 4 (IRF4) and MYC pro-survival signaling (65). In a multicenter, single-arm phase II clinical trial conducted in France, lenalidomide in combination with rituximab was effective in patients with R/R PCNSL, including ocular lymphoma, resulting in remission rates of up to 70%, median PFS of approximately eight months, and OS of approximately 19 months (66). In a small study, ten patients with R/R CNSL, in which 5-10 mg lenalidomide was used as maintenance therapy after first-line salvage therapy, maintained CR for >2 years. This suggests that lenalidomide could be a potential maintenance or consolidation therapy drug (67).

### 4.2 Pomalidomide

Pomalidomide, a third-generation immunomodulatory agent, was approved for marketing by the FDA in March 2013 for multiple myeloma (MM) (68). It was launched in China in November 2020. Pomalidomide has been demonstrated to cross the BBB. Although this small molecule’s chemical structure and mechanism of action are similar to those of the previous generations of dopamine, pomalidomide has more potent efficacy and better safety than lenalidomide. Moreover, it shows no cross-resistance with lenalidomide, low kidney metabolism, and fewer side effects. Also, there is no dose adjustment needed for patients with myeloma with renal loss (except for patients requiring dialysis). More importantly, pomalidomide is still effective in myeloma patients who have failed treatment with lenalidomide and thalidomide and who are resistant to other new drugs.

Pomalidomide has a broad mechanism of action, and so far, 227 clinical studies have been executed for myeloma, brain tumors, Kaposi’s sarcoma, and hematologic tumors. However, there are only three clinical trials of pomalidomide for PCNSL (NCT01722305, NCT03798314, NCT01421524). Among them, NCT01722305 investigated its safety and efficacy for PCNSL in combination with dexamethasone. Out of enrolled 25 subjects with an ORR of 48% (12/25), six patients achieved CR, two patients unconfirmed CR (CRu), and four patients PR, with an overall median PFS of 5.3 months and PFS of nine months for those with effective treatment (69). Notably, one of the patients developed pseudoprogression after four cycles of treatment. CSF analysis determined the CSF-to-plasma ratio of pomalidomide as 19%. Grade 3 or 4 drug toxicity reactions with pomalidomide therapy included hematologic events (neutropenia in 21% and thrombocytopenia in 8%) and nonhematologic events (pulmonary infections in 12%, fatigue in 8%, syncope in 4%, sepsis in 4%, respiratory failure in 8%, and rash in 4%).

## 5 Immune checkpoint inhibitors

Immune checkpoint inhibitors (ICIs) are used to destroy tumor cells by inhibiting the immune checkpoint to turn off the escape mechanism of tumor cells and reactivate the immune cells (70).ICIs enhance T lymphocytes’ proliferation, migration, and cell-killing activity. The three main ICI drug targets are CTLA4, PD-1, and PD-L1. There are few reports about anti-CTLA4 and anti-PD-L1 mAbs for PCNSL and no ongoing clinical trials of anti-CTLA4 mAbs (71). Two clinical trials of anti-PD-L1 mAbs for PCNSL were initiated. However, one was terminated due to poor outcomes, and the other was withdrawn for unknown reasons.

Four et al. studied 32 PCNSL specimens and found that PD-1 expression in tumor-infiltrating lymphocytes was 58% and PD-L1 expression in tumor cells was 37%, while patients with high PD-1 expression had significantly lower OS than patients with low PD-1 expression (71). Similarly, Berghoff et al. observed PD-1 and PD-L1 expression in PCNSL cells and the tumor microenvironment. Moreover, genetic studies have identified high-frequency copy number alterations of the 9p24.1/PD-L1/PD-L2 gene fragment in PCNSL samples (72).

The structural underpinnings of PCNSL immune evasion and the characterization of an inhibitory tumor microenvironment support the use of ICIs in treating PCNSL (21). Encouraging initial results were achieved with the anti-PD-1 antibody nivolumab in four patients with R/R PCNSL and one with CNS-relapsed testicular lymphoma (73). Nivolumab was well tolerated, resulting in four CRs and one PR, of which three patients remained progression-free at 13 to 17 months. Another single-center retrospective study, including eight patients with PCNSL treated with nivolumab, also reported high ORR, with three subjects achieving CR and four achieving PR (74). In a phase 2 study, 27 PCNSL patients were treated with sintilimab combined with high-dose methotrexate, temozolomide, with an ORR of 96.3%, of which 25 achieved CR, and PFS and OS were not reached. The most common grade 3-4 treatment-related toxicities were increased levels of alanine aminotransferase (17.9%) and aspartate aminotransferase (14.3%) (75). There are currently five ongoing clinical trials on PD-1 for PCNSL, as shown in **Table 3**.

**Table 3:** Ongoing clinical trials on PD-1 for PCNSL.

Registration No.	Type	Study protocol	Cycle	No. of people	From - To	Key outcome indicators
NCT04831658	Phase II	“3 + 3” dose-escalation	21-day cycle, six cycles in total	40	Mar 2021 -Sept 2024	DLT, PFS, OS
NCT04899427	Phase II	Orelabrutinib 150 mg/day, siltuximab 200 mg, or tislelizumab 200 mg given on D1 of each cycle	One cycle of 21 days, six cycles in total	32	Mar 2021 -Oct 202	ORR
NCT02779101	Phase II	Pembrolizumab 200 mg	Every 3 weeks	21	June 2016 -June 2019	Efficacy assessment according to IPCG
NCT03255018	Phase II	Pembrolizumab 200 mg	Every 3 weeks until progression or intolerable drug toxicity	12	Feb 2018 -July 2023	ORR
NCT05211336	Phase I	D1 nivolumab 360 mg, lenalidomide 10 mg, or 15 mg (D1-14) in a 21-day cycle. Subsequently, VIPOR treatment (vinblastine, ibrutinib, prednisone, obinutuzumab, and lenalidomide) for one cycle of 21 days.	21-day cycle, six cycles in total	12	April 2022-Jun 2028	Safety
NCT05425654	Phase II	4 cycles of RL-MPV (rituximab, lenalidomide, methotrexate, procarbazine, vincristine) as induction, then ASCT. After 3 months after ASCT, maintenance therapy with nivolumab 3 mg/kg every 2 weeks for 6 months.	cycles of 14 days	30	May 2021 -May 2026	frequency of adverse events, response rates
NCT04158128	Observational	–	–	100	Oct 2016 -Dec 2022	OS, PFS

D, day; DLT, dose-limiting toxicity; IPCG, International PCNSL Collaborative Group; ORR, objective remission rate; ASCT, autologous stem cell transplantation; OS, overall survival; PFS, progression-free survival. Clinical study data were obtained from the ClinicalTrials.gov database.

## 6 Cellular immunotherapy

Many clinical trials have demonstrated that CAR-T cells targeting the pan-B cell antigen CD19 have significant therapeutic effects in the treatment of DLBCL, acute B-lymphocytic leukemia, and MCL (76). CAR-T has become a rising star in the treatment of hematologic cancers. In clinical trials of CAR-T therapy, PCNSL patients are usually excluded due to the potential serious adverse effects, such as cytokine release syndrome (CRS), CAR-T-related encephalopathy syndrome (CRES), and fatal adverse effects when applied in CNS lesions (20). However, due to the advances in CAR-T therapy and the improvements in CAR-T preparation techniques and design in recent years, PCNSL patients have also started to be included in clinical trials of CAR-T therapy. Given that almost all CNSL patients have CD19 expression on malignant B cells, and intravenous CAR-T targeting CD19 (CD19CAR) cells can migrate from the periphery into the CNS and be detected in the CSF, CAR-T therapy may provide a promising treatment option for CNSL patients (20).

### 6.1 CAR T-cell therapy for CNSL

To date, clinical data on CAR-T efficacy in CNSL are scarce, with only five studies reporting clinical outcomes of CAR-T for PCNSL/SCNSL, all using a CD19-CAR (**Table 4**). The first clinical trial (TRANSCEND-NHL-001) by Abramson et al. (77) reported a special case of a 68-year-old female with refractory DLBCL who received CAR-T product lisocabtagene maraleucel (formerly known as JCAR017). After successful T-cell isolation and lymphocyte depletion before CAR-T treatment, the investigators reassessed the patient’s lesion staging, at this point, a new right temporal mass was identified consistent with a diagnosis of SCNSL. Nevertheless, the patient received lymphocyte depletion treatment and intravenous CAR-T infusion as planned. The lesions in the brain regressed, and the patient achieved CR one month after the end of the infusion. At the time of the case report publication, the patient had sustained CR for 12 months without adverse events such as CRS and CRES. In 2017, the FDA approved another CD19-CAR product, tisagenlecleucel (formerly CTL019), with an indication for SCNSL. Accordingly, a retrospective cohort study was reported that included eight patients with SCNSL (sites involved included brain, spinal cord, and meninges) who received lymphocyte depletion treatment and a single dose of intravenous infusion of tisagenlecleucel (78). These patients experienced only mild neurotoxicity or systemic adverse effects, none requiring pharmacological intervention. The 28-day post-treatment outcome assessment showed CR in two patients, PR in two patients, and disease progression in four patients (including two deaths). The 90-day outcome assessment revealed sustained control of lesions in three out of four patients who initially responded to CAR-T therapy and sustained CR in one patient who remained in CR at day 180. Recently, Siddiqi et al. reported significant anti-tumor effects of CAR-T therapy in CNS lesions in a still ongoing prospective clinical trial of CD19-CAR for B-cell NHL (NCT02153580) (79). The CAR-T cell product was modified to express a truncated human epidermal growth factor receptor (eGFR), which can act as an antibody target to rapidly clear CAR-T cells *in vivo* in cases of severe toxicity from CAR-T therapy. In the preliminary study, three PCNSL and four SCNSL patients received an intravenous infusion of CAR-T product. No severe life-threatening adverse reactions were observed in any of the patients, with the additional application of the anti-IL-6 receptor antagonist tocilizumab mAb for intervention in two cases of CRS and steroid hormone for intervention in three cases of CRES. Four patients responded to the treatment, with one patient achieving CR and three patients PR; however, it is still being determined whether these responses will be durable due to a short follow-up time of only a few weeks.

**Table 4:** Summary of study results of CAR-T for PCNSL/SCNSL.

Author	Study design	Study subjects	Route of administration	Antigen selection	Adverse effects	Outcome	NCT/ ChiCTR
Abramson et al. (74)	Case report of a patient included in a phase I clinical trial	SCNSL (n=1) DLBCL	Intravenous	Lisocabtagenemaraleuce (JCAR017): CD19CAR	Not present	Show as CR after 1 month	NCT02631044
Frigault et al. (78)	Retrospective cohort study	SCNSL (n=8) DLBCL (n=5) high-grade B cell lymphoma (n=2) mediastinal B cell lymphoma (n=1)	Intravenous	Tisagenlecleucel: CD19CAR	Grade 1 CRS (n=7) No neurotoxicity No need for tocilizumab or steroids	PD after 3 days (n=4) and after 25 days (n=2) PR (n=2), one maintained for 90 and the other for 180 days, CR (n=2), one maintained for 90 and the other for 180 days,	NCT04134117
Siddiqi et al. (79)	Preliminary results of an ongoing Phase I clinical trial	PCNSL (n=3) SCNSL (n=4)	Intravenous (n=7); intracerebroventricular	CD19CAR modified to express truncated eGFR	Grade 1-2 CRS and neurotoxicity, steroid intervention (n=2) or tocilizumab intervention (n=3)	CR (n=1) PR (n=3)	NCT02153580
Li et al. (80)	Phase I clinical trial	PCNSL (n=1) SCNSL (n=4)	Intravenous	CD19CAR and CD22CAR in combination	Presence of grade 1 (n=4) and grade 2 (n=1) CRS. Presence of grade 1 (n=1) and grade 4 (n=1) neurotoxicity requiring steroids, plasma replacement, tocilizumab intervention	CR (n=1), PR (n=4) at 60-day assessment	ChiCTR- OPN- 16008526
Frigault et al. (78)	Phase I/II clinical trial	12 cases of R/R PCNSL	Intravenous	Tisagenlecleucel: CD19CAR	Level 1 CRS (58.3%), Low-level ICANS (41.6%), Level 3 ICANS (8.3%)	CR (n=6), total effective number (n=7)	NCT04134117

CR, complete remission; CRS, cytokine release syndrome; ICANS, immune effector cell-associated neurotoxicity syndrome; DLBCL, diffuse large B-cell lymphoma; R/R PCNSL, relapsed/refractory primary central nervous system lymphoma; PD, progressed disease; SCNSL, secondary central nervous system lymphoma.

Notably, Li et al. from Tongji Hospital in China reported the efficacy of dual antigen-specific CAR T therapy, including one case of PCNSL and four cases of SCNSL that received CD19-CAR and CD22-CAR treatment (CD22 is another pan-B cell target that could enhance the treatment potency in case of CD19 antigen loss) (80). In this cohort, one patient developed mild neurotoxic symptoms and one patient severe neurotoxic symptoms requiring steroid and plasma replacement therapy. All patients experienced remission within 60 days of treatment, including two CRs. However, four patients relapsed within three to eight months of follow-up, with a median PFS of three months. Despite tumor recurrence, the analysis of tumor tissue and CSF in one of the patients revealed persistent expression of target tumor antigens and detectable CAR-T cell infiltration. The authors hypothesized that the tumor immunosuppressive microenvironment may influence CAR-T cell action, leading to tumor recurrence. Subsequently, Frigault et al. investigated the efficacy and safety of tisagenlecleucel in R/R PCNSL. They determined its overall efficacy rate as 58.3% and a CR rate of 50%, with three patients achieving and maintaining CR without any life-threatening events or treatment-related deaths (78).

The above clinical trial results indicate that the safety of CD19CAR therapy for CNSL is moderate. However, better therapeutic effects are achieved when managing the adverse effects under control. Therefore, several phase I/II clinical trials have gradually emerged to investigate further the safety and efficacy of CD19-CAR application in SCNSL and PCNSL (**Table 5**).

**Table 5:** Ongoing clinical trials of CD19-CAR for PCNSL/SCNSL.

Registration No.	Type	Study protocol	Cycle	No. of people	From - To	Key outcome indicators
**NCT04443829**	Phase I	C1 with 250 x 10^6 CD19 CAR T-cells i.v. following LD chemotherapy C2 with 25 x 10^6 CD19 CAR T-cells intraventricularly via an Ommaya reservoir following LD chemotherapy	2	12	March 2021 - Dec 2032	1. Feasibility of generating the CD19CAR T-cell 2. Safety of administration 3. Efficacy
**NCT04608487**	Phase I	CD19-CAR fludarabine and cyclophosphamide	1	18	Dec 2020 - June 2025	1. Number of participants with Treatment-related AEs 2. ORR, CR, DOR, PFS, OS
**NCT04464200**	Phase I	A standard 3 + 3 dose escalation design. CAR-T cells by IV infusion, depending on the dose level and formulation of the final CAR-T cells	1	30	July 2020 - July 2026	1. Recommended phase II dose 2.ORR
**NCT03484702**	Phase II	Application of CD19-CAR after lymphatic depletion	Not specified	113	Sep 2018 - Dec 2023	1. ORR, AEs 2. Incidence of Aes, OS, PFS, ORR, CRR, DOR, EFS
**NCT04532203**	Phase I	A standard 3 + 3 dose-escalation design. A total of 3 dose levels are set for subjects.	Not specified	72	Nov 2020 - Nov 2026	1. DLT, incidence of TEAEs 2. ORR, OS, event-free survival, quality of life

PCNSL, relapsed/refractory primary central nervous system lymphoma; SCNSL, secondary central nervous system lymphoma; C, cycle; CR, complete remission; D, day; OS, overall survival; PFS, progression-free survival. LD, lymphodepletion; ORR, overall response rate; AEs, adverse events; CRR, complete response rate; EFS, event-free survival; DOR, duration of response; DLT, dose-limiting toxicity; TEAEs, treatment-emergent adverse events. Clinical study data were obtained from the ClinicalTrials.gov database.

In addition, antibodies with improved structure and dual-specificity have been invented to enhance the patient’s immune response to CAR-T therapy. Bispecific T-cell inducers (BiTEs) are engineered bispecific mAbs with two single-chain variable structural domains of different antibodies. One structural domain targets the CD3 receptor on T cells, while the other targets a tumor-specific antigen (81). BiTEs link T cells and tumors, triggering cell death by target cell lysis without conventional major histocompatibility complex (MHC) class I/peptide antigen recognition (82). Currently, there is only one ongoing Phase I/II study of bispecific Anti-CD19 anti-CD20 CAR-T cells for patients with PCNSL (NCT04186520). In 2023, Zou et al. reported a patient with multiline-resistant refractory PCNSL who received decitabine-primed tandem CD19/CD22 CAR-T therapy with PD-1 and BTK Inhibitors maintenance and eventually maintained complete remission (CR) for a 35-month follow-up period (83). Besides improving the CAR structure, alternative cell lines with antigen specificity, such as CAR-NK cells and CAR-macrophages, have been developed, demonstrating enhanced anti-tumor anti-tumor activity (84). Furthermore, phase I/II clinical trials have shown fewer adverse effects with CAR-NK therapy compared to regular CAR-T therapy in treating systemic lymphoma (85). Despite that, more clinical efficacy data is still needed before it might be considered a future therapeutic strategy for patients with PCNSL.

### 6.2 Challenges of CAR-T therapy for CNSL

#### 6.2.1 CNSL has immune escape tumor characteristics

Tumor-associated macrophages/microglia (TAM/M) are essential to non-tumor cells in PCNSL, providing an immunosuppressive microenvironment for tumor cell migration, survival, and expansion (86). It has been shown that in the PCNSL microenvironment, in addition to the secretion of immunosuppressive cytokines, tumor-promoting growth is dominated by M2-type TAM/M differentiation, which is correlated with poor tumor prognosis (87). The existence of some TAM subsets can lead to antigen-specific T-cell dysfunction and failure of CAR-T cell therapy (80, 88).

#### 6.2.2 BBB

In intracranial tumors, in addition to the metabolic barrier of the tumor itself that affects CAR-T infiltration, the BBB also limits therapeutic efficacy. Even so, it has been demonstrated that intravenously injected CAR-T cells could cross the BBB and be detected in the brain parenchyma and CSF, but in much lower numbers than in the body circulation (78). Animal studies have shown that intravenous CAR-T therapy administration had an anti-tumor anti-tumor effect on PCNSL but was less effective than local administration. Clinical trials have evaluated local CAR-T infusion’s feasibility, safety, and efficacy (directly into the tumor cavity or CSF) for treating primary brain tumors such as glioblastoma (except PCNSL) (89). Hence, local application of CAR-T may be an alternative treatment for PCNSL, as applied in some clinical trials.

#### 6.2.3 Antigen loss

Loss or downregulation of tumor target antigens after treatment may also cause CAR-T treatment failure (90). For example, in patients with B-lymphocytic leukemia, CD19-negative tumor cell relapse occurred in 7-25% of patients. A similar situation has been observed in some cases after CAR-T treatment of systemic lymphoma; presumably, the same problem may exist in CNSL (90). This needs to be further investigated and may require taking multiple biopsies of relapsed CNSL patients to perform real-time antigen analysis of the lesions to adjust to the therapeutic targets at a desirable time.

## 7 Challenges and opportunities for PCNSL treatment

### 7.1 Challenges

The development and application of new drugs and therapies for PCNSL have been complex. The principal causes for this are PCNSL, a rare disease, it is challenging to conduct head-to-head studies with a large sample size, and patient enrollment in clinical trials lasts significantly longer than other malignancies. In addition, many targeted therapeutic agents, such as proteasome inhibitors, are too large to penetrate the BBB. Drug BBB permeability remains a challenge in the treatment of CNS malignancies, including PCNSL. Significantly, the intracerebral drug concentration directly determines whether the killing of tumor cells can be achieved.

The treatment of elderly and frail patients who cannot tolerate chemotherapy is also gaining considerable attention (91). Since the vast majority of R/R PCNSL patients are older than 60 years old, their physical functions decrease, and often they cannot tolerate chemotherapy or radiotherapy. Autologous stem cell transplantation also tends to exclude elderly and frail patients.

In addition, drug resistance is also a challenge that must be faced in the treatment of PCNSL (92). It includes the resistance of R/R PCNSL to chemotherapy, single-targeted drug therapy, or immunotherapy. Resistance to anticancer drugs arises not only from genetic mutations that occur at the initial diagnosis of the tumor but also from genetic mutations and epigenetic modifications, such as loss of tumor antigens, that may be induced during treatment.

### 7.2 Opportunities

It is possible to attain increased intracerebral drug concentration by ultrasound or osmotic disruption of the BBB. A multicenter study conducted to assess the disruption of the BBB by using intra-arterial injection of mannitol followed by intra-arterial MTX treatment resulted in an ORR of 81.9%, a CR rate of 57.8%, and an OS of 3.1 years (93). This compares favorably with historical controls, especially considering that approximately half of the patients enrolled in the study did not receive consolidation therapy. In another study for PCNSL, an intravenous infusion of low-dose tumor necrosis factor-α (TNF-α) was used to destroy the BBB, followed by an R-CHOP regimen (rituximab/cyclophosphamide/doxorubicin/vincristine/prednisone), and the patients had a reasonable response rate (94).

Besides, nanomedicine has emerged as a promising approach to overcome BBB limitations, with various nanoparticle formulations being developed to enhance drug delivery to brain tumors (95, 96). Nanoparticles, including those functionalized with therapeutic agents like rituximab, have demonstrated improved targeting and therapeutic effects in preclinical models (97, 98).

Furthermore, targeted and immunotherapy may play an essential role in improving the treatment for this patient group compared to chemotherapy and radiotherapy because of the low incidence of side effects and relatively better tolerability.

Finally, combination studies are an effective strategy to reduce drug resistance. For example, BTK inhibitors have limited efficacy and short maintenance when used as monotherapy, but when used in combination with chemotherapy and immunotherapy, a more durable response can be achieved. Our studies (49) demonstrated this using ibrutinib monotherapy and combination therapy. Several studies exploring combination therapy are also underway, and it remains to be seen whether combination treatment strategies will improve the efficacy of treatment for PCNSL and the long-term prognosis of patients.

## 8 Future directions

With the improved understanding of the molecular pathology of PCNSL and the progress in targeted therapeutic strategies, the development of minimally invasive biomarkers is becoming possible. qPCR, ddPCR, and second-generation sequencing have driven the growth of liquid biopsies for diagnostics of PCNSL (99). Studies have shown that the mutation profile of circulating tumor DNA (ctDNA) overlaps with the gene mutation profile of tumor tissue. The presence of ctDNA has been detected in the CSF, blood, and urine of PCNSL patients, with a high detection rate of mutations even in urine, suggesting that liquid biopsy using plasma or urine may be a less invasive alternative in diagnosing PCNSL (100). The abundance of ctDNA has been associated with the tumor load and thus could predict treatment response and disease prognosis (53, 101). A prospective study is currently underway to determine whether the detection of ctDNA in CSF has prognostic significance for predicting treatment response (NCT04401774). Compared to other intracranial malignancies, PCNSL has some characteristic genetic features, such as *MYD88* and *CD79B* mutations, and ctDNA may also play a role in the diagnosis and differential diagnosis of PCNSL (102).

The efficacy of targeted therapies and immunotherapy in PCNSL is impressive yet expected. They have the potential to change the treatment options for PCNSL, and chemotherapy-free treatment approaches are currently lined up for patients with primary PCNSL. Moreover, targeted therapies and immunotherapy can potentially become (part of) the first-line treatment regimen. They are good options for relapsed, refractory, elderly, and frail patients who cannot tolerate radiotherapy. In addition, these new treatments have fewer toxic side effects, including adverse effects on cognitive function, than traditional therapies. The combination of chemotherapy, targeted therapy, and immunotherapy is expected to improve the prognosis of PCNSL patients and improve their quality of life.
